# Case Report: A Case of β-Ureidopropionase Deficiency Complicated With MELAS Syndrome Caused by *UPB1* Variant and Mitochondrial Gene Variant

**DOI:** 10.3389/fped.2022.838341

**Published:** 2022-02-21

**Authors:** Jianbo Shu, Xiufang Zhi, Jing Chen, Meifang Lei, Jie Zheng, Wenchao Sheng, Chunhua Zhang, Dong Li, Chunquan Cai

**Affiliations:** ^1^Tianjin Children's Hospital (Children's Hospital of Tianjin University), Tianjin, China; ^2^Tianjin Pediatric Research Institute, Tianjin, China; ^3^Tianjin Key Laboratory of Birth Defects for Prevention and Treatment, Tianjin, China; ^4^Graduate College of Tianjin Medical University, Tianjin, China; ^5^Department of Radiology, Tianjin Children's Hospital, Tianjin, China; ^6^Department of Neurology, Tianjin Children's Hospital, Tianjin, China; ^7^MILS International, Yokohama, Japan; ^8^Department of Neurosurgery, Tianjin Children's Hospital, Tianjin, China

**Keywords:** β-ureidopropionase deficiency, *UPB1* gene, MELAS syndrome, mitochondrial DNA, whole-exome sequencing

## Abstract

**Background:**

β-Ureidopropionase deficiency is a rare autosomal recessive disease affecting the last step of pyrimidine degradation. Mitochondrial encephalomyopathy with lactic acidosis and stroke-like episodes (MELAS) syndrome is a rare inherited disorder caused by genetic defects in mitochondrial DNA.

**Case Presentation:**

One 8-year-old boy presented with dizziness, vomiting, and convulsions. The gas chromatography–mass spectrometry results suggested β-ureidopropionase deficiency. The whole-exome sequencing results revealed homozygous missense variant c.977G>A (p.R326Q) in *UPB1*. However, the patient presented with persistent hyperlactacidemia and metabolic acidosis, which did not correspond to the classic features of β-ureidopropionase deficiency. Combined with the manifestations of developmental delay, poor academic performance, and poor sports stamina, whole-mitochondrial-genome sequencing was performed. The results exhibited the variant m.3243A>G of *MT-TL1* gene. The level of heterogeneity was 65% in the patient and 17.8% in his mother. Eventually, the final diagnosis of β-ureidopropionase deficiency combined with MELAS syndrome was made.

**Conclusion:**

The report about β-ureidopropionase deficiency caused by a nuclear gene variant and MELAS syndrome caused by a mitochondrial gene variant coexisting in the same patient enriches the clinical study of these two rare diseases.

## Introduction

β-Ureidopropionase deficiency caused by *UPB1* variant is a rare autosomal recessive disease affecting the last step of pyrimidine degradation. *UPB1* is located on chromosome 22q11.2 and consists of 10 exons, encoding a protein of 384 amino acids. Currently, there have been approximately 30 genetically confirmed cases reported worldwide (1–4). The phenotype is highly variable, ranging from being symptom-free to a severe neurological illness (1, 2, 5, 6).

Mitochondrial encephalomyopathy with lactic acidosis and stroke-like episodes (MELAS) syndrome is a rare inherited disorder caused by genetic defects in mitochondrial DNA and is characterized by fluctuating encephalopathy, migraine, seizures, and stroke-like episodes. MELAS syndrome is the most common subtype of mitochondrial encephalomyopathy and mainly associated with m.3243A>G variant of *MT-TL1* gene which encodes the mitochondrial transfer ribonucleic acid leucine-1. In 80% of cases, MELAS syndrome is due to the variant m.3243A>G (7). Typical brain imaging features of MELAS syndrome include stroke-like areas, calcification of the basal ganglia, and brain atrophy.

With the development of sequencing technology, it is possible to study human gene variant by DNA sequencing. Whole-exome sequencing (WES), capable of sequencing all protein-coding regions of the human genome (exome), is rapidly becoming the most widely used targeted enrichment method, especially for monogenic diseases. Exonic variants have been shown to be responsible for most monogenic diseases, with missense and nonsense variants alone accounting for about 60% of the disease variants (8, 9). Exons account for about 1.5% of genomic DNA and contain about 22,000 genes (10). Targeted sequencing of about 1.5% of the human genome as an effective way to discover human pathogenic/likely variants mainly rests on the fact that about 85% of variants in exons have a great influence on disease-related traits (11). Recent studies have shown the benefits of WES in early diagnosis of epilepsy and other neurodevelopmental disorders, but WES only focuses on nuclear genomes, not mitochondrial genes. Therefore, mitochondrial DNA analysis becomes critical for finding pathogenic variants in mitochondrial genes leading to the related disease.

Here we report a rare case of β-ureidopropionase deficiency caused by *UPB1* gene variant complicated with MELAS syndrome caused by a mitochondrial gene variant, which has not been previously described.

## Case Presentation

One 8-year-old boy from a non-consanguineous Chinese family was admitted to the hospital in September 2018 because of dizziness, vomiting, and convulsions. Dizziness occurred 1 day before admission, accompanied by blurred vision. The child had convulsions twice within 18 h and presented with generalized tonic–clonic seizures without fever. The development of the patient was delayed for more than 2 years. His height was 113 cm (−2 SD). He was born at term by cesarean section due to the scarred uterus of his mother, with a weight of 3,650 g (G3P2). No history of postnatal asphyxiation was reported. His father had hyperuricemia for more than 10 years. The height of his mother was 146 cm. The family had no history of genetic/metabolic diseases or congenital disorders.

**Figure 1:**
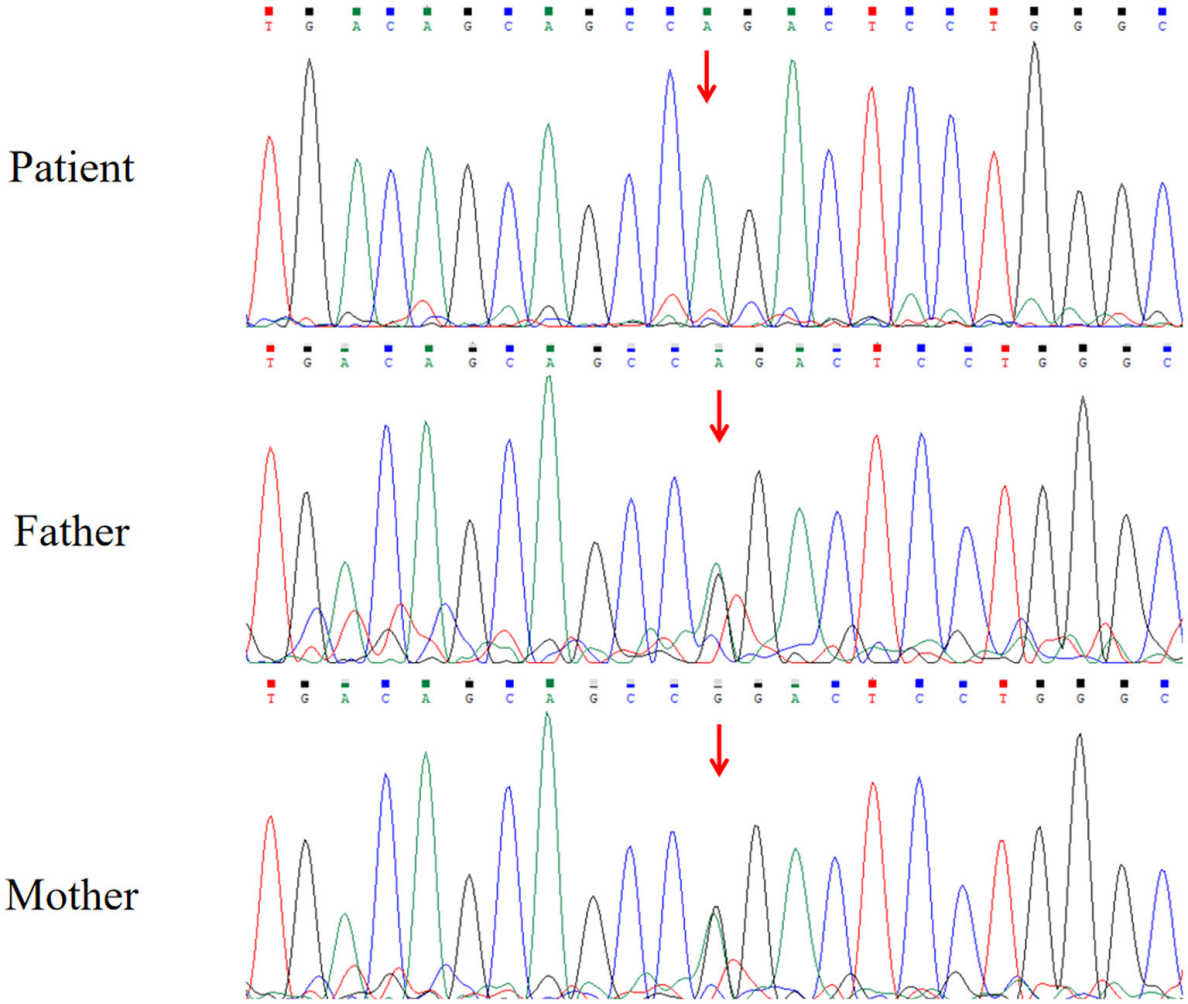
Results of Sanger sequencing.

The brain magnetic resonance imaging (MRI) results showed no specific findings. The electroencephalogram (EEG) showed occipital slowing during awake state. His serum level of lactate was 7.04 mmol/L (range: 0.5–2.2 mmol/L), and the cerebrospinal fluid level of lactate was 4.63 mmol/L. Increased pyrimidine and metabolite levels in urine were detected by gas chromatography–mass spectrometry (GC–MS), and β-ureidopropionase deficiency was suspected (Table 1). The analyses of karyotype by G-banding showed a normal male 46, XY karyotype. The analysis of WES showed that the patient had a homozygous missense variant c.977G>A in exon 9 of *UPB1* (NM_016327.3), leading to the amino acid substitution p.R326Q. Sanger sequencing showed that both of his parents carried a heterozygous variant of c.977G>A (Figure 1). The parents of the patient refused the whole-mitochondrial-genome sequencing.

**Table 1:** Pyrimidine degradation metabolites of the patients.

**Metabolites**	**Patient**	**Control range**
Uracil	0.0660	<0.41
Thymine	0.0328	<0.003
Dihydouracil	0.1297	<0.001
Dihydothymine	0.1297	<0.001
N-Carbamyl-β-alanine	0.1722	<0.001
N-Carbamyl-β-aminoisobutyric acid	0.6600	<0.001

*Value: peak area ratio to creatinine*.

In the following 2 months, convulsions occurred once for each of them, and the manifestations were hanging of eyes, cyanosis of the lips, head backward, and stiffness of limbs accompanied by rhythmic tremor, without fever. The IQ assessments suggested that his intelligence was below average. The level of serum lactate was 15.16 mmol/L. Blood–gas analysis indicated metabolic acidosis (decompensated). Because the patient had manifestations of developmental delay, poor academic performance, poor sports stamina, hyperlactacidemia, and metabolic acidosis, mitochondrial disease was further suspected. Whole-mitochondrial-genome (mtDNA; RefSeq NC_012920) sequencing (2019.8) of peripheral blood sample was performed. It revealed a variant m.3243A>G in *MT-TL1* gene, and the proportion of sequences containing the variant was 65% in blood cells. The same variant was detected in his mother, with 17.8% heterogeneity in blood. The diagnosis of MELAS syndrome was established.

At 7 months later, the child developed paroxysmal visual impairment and was readmitted to the hospital in January 2021. At 3 days before admission, the child developed paroxysmal blurred vision, which lasted for several minutes each time. The vision was normal after spontaneous remission, accompanied by severe swelling and pain in the posterior occipital region, vomiting twice, and no ejection. The physical examination showed left central facial palsy. The right upper limb and lower limb muscle strength was level 4, and the left upper limb muscle strength was level 3. The muscle tension of the left ankle was slightly higher than that of the right. Bilateral Babinski signs were suspected positive.

**Figure 2:**
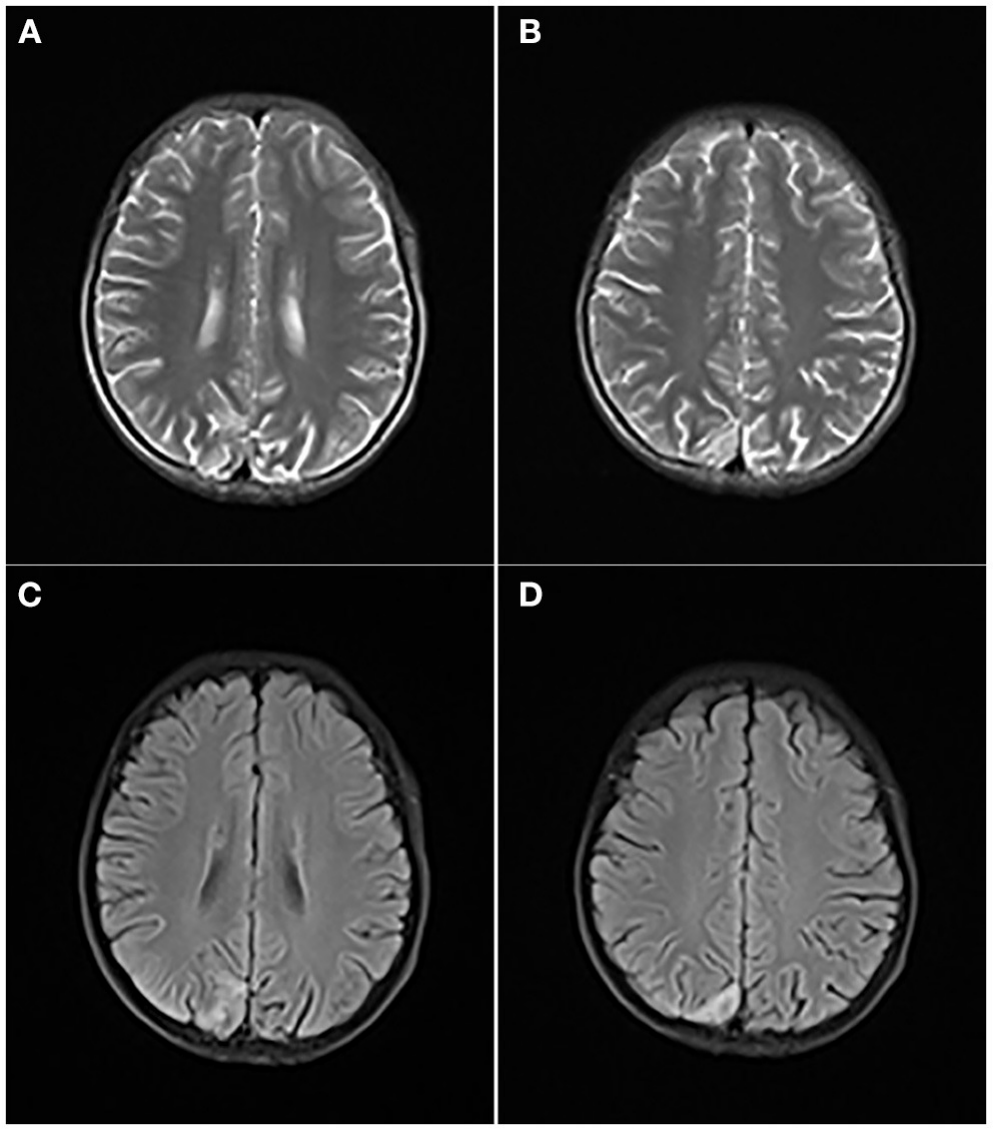
T2WI **(A,B)** and FLAIR **(C,D)** revealed a high signal in the right parietooccipital cortex and subcortical region, in addition to widened bilateral cerebellar hemispheres and bilateral parietooccipital sulci.

A computed tomography of the head demonstrated a small, patchy, low-density shadow in the right occipital part and a slightly high-signal shadow in the left basal ganglia. On the day of admission, he had convulsions for 3 times, presenting as a left focal generalized systemic attack, which lasted for 5–10 s to relieve. The brain MRI revealed a high signal in the right parietooccipital cortex and subcortical region about T2WI (Figures 2A,B) and FLAIR (Figures 2C,D) sequence, in addition to widened bilateral cerebellar hemispheres and bilateral parietooccipital sulci. Ambulatory EEG showed abnormal EEG in children, with background slow-wave activity. The electrophysiologic study showed that there was no abnormality in the electroneurogram of both lower limbs, and myogenic damage was shown in electromyography. Brainstem auditory-evoked potential test indicated bilateral peripheral abnormalities with moderate hearing loss of his right ear and mild hearing loss on the left. The visual-evoked potential test indicated an abnormal bilateral visual pathway. The level of ammonia in blood was temporarily elevated with a value of 137 μg/dl. Hyperlactacidemia and metabolic acidosis persisted but improved.

He was treated with topiramate for seizure control, and his epilepsy was well controlled. Then, the patient received the cocktail therapy and supportive treatment for MELAS syndrome. What is more, he was not allowed to use drugs that can affect mitochondrial function. Later, the symptoms of the child improved, and he was followed up for 3 months after discharge.

## Discussion

Our case had a tortuous process of diagnosis. At the beginning, the child developed symptoms such as growth retardation and convulsions, which were suspected to be indicative of a genetic metabolic disease. Urine metabolic screening by GC–MS suggested a deficiency of β-ureidopropionase. Furthermore, WES was performed, and a homozygous pathogenic variant of *UPB1* gene was found, which confirmed β-ureidopropionase deficiency. In the clinical examination, we found that the blood lactate level of the child was elevated, but his parents refused the mitochondrial DNA analysis. Although no related abnormalities were found in the head MRI, there were hyperlactacidemia and metabolic acidosis in the course of the disease, which were not consistent with the classic features of β-ureidopropionase deficiency. Given the combination of developmental retardation, poor academic performance, and poor exercise tolerance, a mitochondrial disease was suspected, and a mitochondrial DNA analysis of peripheral blood sample was finally performed to diagnose MELAS syndrome. Later in the course of the disease, the brain MRI of the patient also showed abnormalities, suggesting that the absence of abnormalities in the brain MRI at the beginning may be due to the early stage of the disease. To our knowledge, this is the first case of β-ureidopropionase deficiency complicated with MELAS syndrome.

Our case had a homozygous missense mutation c.977G>A in exon 9 of the *UPB1* gene. The *UPB1* gene encodes β-ureidopropionase, the final reaction enzyme of the uracil and thymine metabolic pathway. This variant causes the conversion of the 326th amino acid of the encoded protein from arginine to glutamine, thus affecting the activity of β-ureidopropionase. Defection of β-ureidopropionase activity directly affects the degradation of N-carbamyl-β-alanine and N-carbamyl-β-aminoisobutyric acid, resulting in an abnormal increase of N-carbamyl-β-alanine and N-carbamyl-β-aminoisobutyric acid in the urine and plasma. At the same time, uracil, thymine, 5,6-dihydrouracil, and 5,6-dihydrothymine, which are the upstream metabolic substrates, will also accumulate in varying degrees, causing a series of clinical symptoms with nervous system abnormalities and developmental abnormalities. All of these metabolites had various elevated levels in the urine of our patient (Table 1). To date, only 19 variants have been reported (HGMD, http://www.hgmd.cf.ac.uk/ac/), including 14 missense variants, 4 splice site variants, and 1 small deletion variant. c.977G>A is the most common variant in the Chinese population. According to the ACMG variant classification criteria, the c.977G>A variant is classified as likely pathogenic (PS3, PP3, and PP4). Recently, a variant in *UPB1* gene, c.977G>A (p.R326Q), was shown to be common in Japanese patients (2). Highly variable phenotypes ranging from early infantile onset with severe neurological involvement to mild developmental delay and learning disabilities to asymptomatic individuals were observed in the diagnosed patients.

MELAS syndrome may overlap with β-ureidopropionase deficiency but shows distinctive imaging features. Our case had no specific MRI findings at the beginning, which led us to fail to diagnose MELAS syndrome early. Patients with MELAS syndrome are usually short and mentally retarded (12). Other common symptoms include headache, vomiting, hemiplegia, blindness, and seizures. Our case was short and mentally retarded, with dizziness, vomiting, convulsions, and stroke-like episodes. Another important manifestation was hyperlactacidemia, which was the key reason for us to suspect mitochondrial encephalomyopathy. The results of the whole-mitochondrial-genome sequencing revealed a variant m.3243A>G in *MT-TL1* gene, which was recognized as the main cause of MELAS syndrome. The proportion of sequences containing the variant was 65% in the child, compared with 17.8% in the mother. His mother was asymptomatic, other than her short stature. A particularly characteristic feature of MELAS syndrome is that recurrence may occur in different locations of the brain than previously noted. Our child only showed brain MRI abnormalities in the most recent course of the disease. Long-term follow-up is required to observe the changes of brain imaging. Furthermore, previous work suggested that the contribution of nuclear genetic background may influence the clinical heterogeneity in m.3243A>G-related classical mitochondrial disease, which highlighted the need to screen nuclear gene variants and function for an improved diagnosis of mitochondrial disorders (13).

At present, WES is the most widely used method to detect mendelian genetic diseases. It can sequence the protein-coding regions (exons) in their entirety, but it cannot reveal the variant of deep intron region and mitochondrial gene. In the diagnosis of clinical cases, it is necessary to select the appropriate detection methods to assist in the diagnosis of diseases according to the clinical situation of the cases. In our case, the child was diagnosed with both β-ureidopropionase deficiency and MELAS syndrome. The β-ureidopropionase deficiency which was caused by *UPB1* variant can be detected by WES, but the MELAS syndrome requires whole-mitochondrial-genome sequencing to be diagnosed.

In conclusion, we suggest that WES should not be used as the only detection method when the clinical phenotype of children is non-specific and genetic metabolic diseases are suspected. In clinical work, it is necessary to combine with the actual situation, especially paying attention to the clinical characteristics that cannot be explained by a single disease, such as hyperlactacidemia and metabolic acidosis in our case, and there is a need to select the appropriate examination methods for auxiliary diagnosis. Our case was diagnosed as β-ureidopropionase deficiency combined with MELAS syndrome, which is very rare.

## Data Availability Statement

The original contributions presented in the study are included in the article/supplementary material, further inquiries can be directed to the corresponding author/s.

## Ethics Statement

Written informed consent was obtained from the minor(s)' legal guardian/next of kin for the publication of any potentially identifiable images or data included in this article.

## Author Contributions

JS and XZ performed the literature search, collection, gene sequencing, and drafting of the manuscript. CZ, JZ, JC, WS, and ML completed all examination and photograph collection and confirmed the final diagnosis. CC and DL performed conceptualization and funding acquisition. All authors read, edited, and approved the final version of the manuscript.

## Funding

This work was supported by the National Natural Science Foundation of China (Grant Number 81771589), the Program of Tianjin Science and Technology Plan (Grant Number 18ZXDBSY00170), and the Public Health and Technology project of Tianjin (Grant Number ZC20120).

## Conflict of Interest

The authors declare that the research was conducted in the absence of any commercial or financial relationships that could be construed as a potential conflict of interest.

## Publisher's Note

All claims expressed in this article are solely those of the authors and do not necessarily represent those of their affiliated organizations, or those of the publisher, the editors and the reviewers. Any product that may be evaluated in this article, or claim that may be made by its manufacturer, is not guaranteed or endorsed by the publisher.
